# Supplementary material for: Bioelectric-calcineurin signaling module regulates allometric growth and size of the zebrafish fin
Source: Sci Rep. 2018 Jul 10;8:10391. doi: 10.1038/s41598-018-28450-6 (PMC6039437; doi:10.1038/s41598-018-28450-6)
Supplement: Supplementary file 1 — Supplementary Information [file 41598_2018_28450_MOESM1_ESM.pdf]

## Supplementary Information:

# Bioelectric-calcineurin signaling module regulates allometric growth and size of the zebrafish fin

Jacob M Daane[1], Jennifer Lanni[1,2], Ina Rothenberg[3], Guiscard Seebohm[3], Charles W Higdon[4], Stephen L Johnson[4], Matthew P Harris[1]

[1]Department of Genetics, Harvard Medical School; Department of Orthopedic Research, Boston Children's Hospital, 300 Longwood Avenue, Boston MA 02115;
[2]Department of Biology, Wheaton College, Norton MA 02766;
[3]Institute for Genetics of Heart Diseases (IfGH), Department of Cardiovascular Medicine, University Hospital Muenster, Muenster, Germany;
[4]Department of Genetics, Washington University Medical School, Saint Louis MO 63110

Corresponding Author
Email: harris@genetics.med.harvard.edu

**Figure S1. Proportion and patterning of zebrafish fins is enhanced by calcineurin inhibition.** Fin growth occurs through sequential addition of a regular pattern of lepidotrichia hemi-ray dermal bone segments at growing end of the fin. Pattering of fin segmentation in A) wildtype, B) *alf* and C) *sof* pectoral fins; stacked blue rectangles model sequential addition and segmentation of individual rays. D) Schematic of regenerating pectoral fin assay to assess relative scaling. One pectoral fin is cut to approximately 50% of original size and allowed to regenerate. Comparisons to the contralateral side enables analysis of previous size and effect of treatment on non-regenerating fins. E) Dose response of FK506 treatment of pectoral fin growth. F-H) Pattern of segmentation of the lepidotrichia in F) regenerating wild-type, or regenerating FK506 treated wild-type or *sof* fins; solid line, plane of resection. While segments are normally restored during regeneration, regenerating fins treated with FK506 show elongated segmentation after regeneration similar to the *alf* phenotype (B). Error bars represent ± SEM. * p-value < 0.05. *** p-value < 0.001.

**Figure S2. Calcineurin inhibition does not re-specify positional information of the caudal fin.** Caudal fins were amputated and grown in FK506 for 15 days. The drug was removed (green arrow indicates stoppage of treatment) at which point the FK506 treated fins ceased further growth. These fins were then re-cut at either the original amputation plane (A) or at the site of the original fin length prior to FK506 treatment (B). Gray dashed line indicates pre-cut fin length. Red dashed lines indicate second resection. Blue dashed lines indicate extent of FK506 overgrowth. Error bars represent ± SEM.

**Figure S3. Deletion screen of *kcnk5b* reveals essential role of C-terminus.** Using specific guide RNAs against the last exon of *knck5b* encoding the last transmembrane and cytoplasmic tail of the channel, we screened injected founders for evidence of overgrowth. We identified localized clones having specific overgrowth of the fins. B) Analysis of the changes in the overgrown tissues demonstrated presence of local deletions in *kcnk5b* predicted to cause truncation of the channel in a comparable location as to the *alf* mutation (C).

**Supplementary Figure S4. Growth deficit during FK506 treatment of juveniles.** Wild-type juvenile zebrafish treated with FK506 did not grow when treated starting at ages of 30 days (A) or 55 days (B). C) Representative image of stunted growth in FK506 treated fish relative to DMSO treated siblings. n=10 fish per group. Error bars represent ± SEM.

# Figure S1

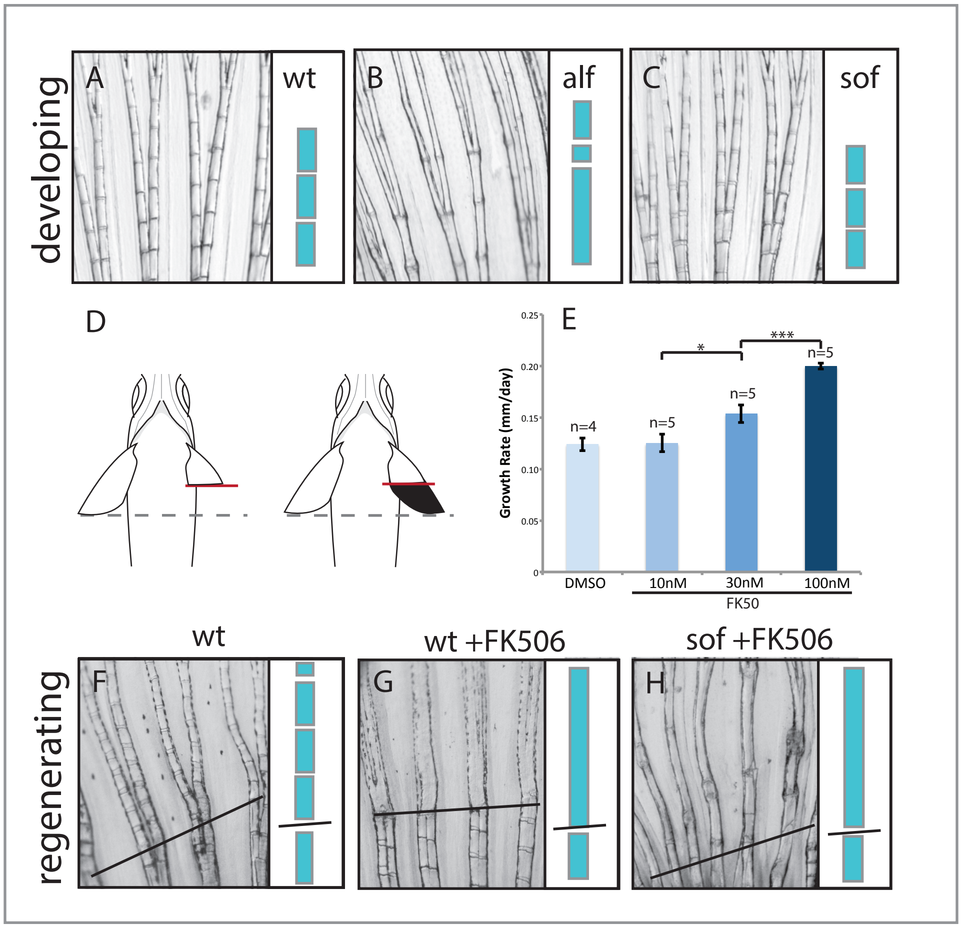

developing
A
wt
B
alf
C
sof
D
E
Growth Rate (mm/day)
0.25
0.20
0.15
0.10
0.05
0
n=4
n=5
n=5
n=5
*
***
DMSO
10nM
30nM
100nM
FK50
regenerating
wt
wt +FK506
sof +FK506
F
G
H

# Figure S2

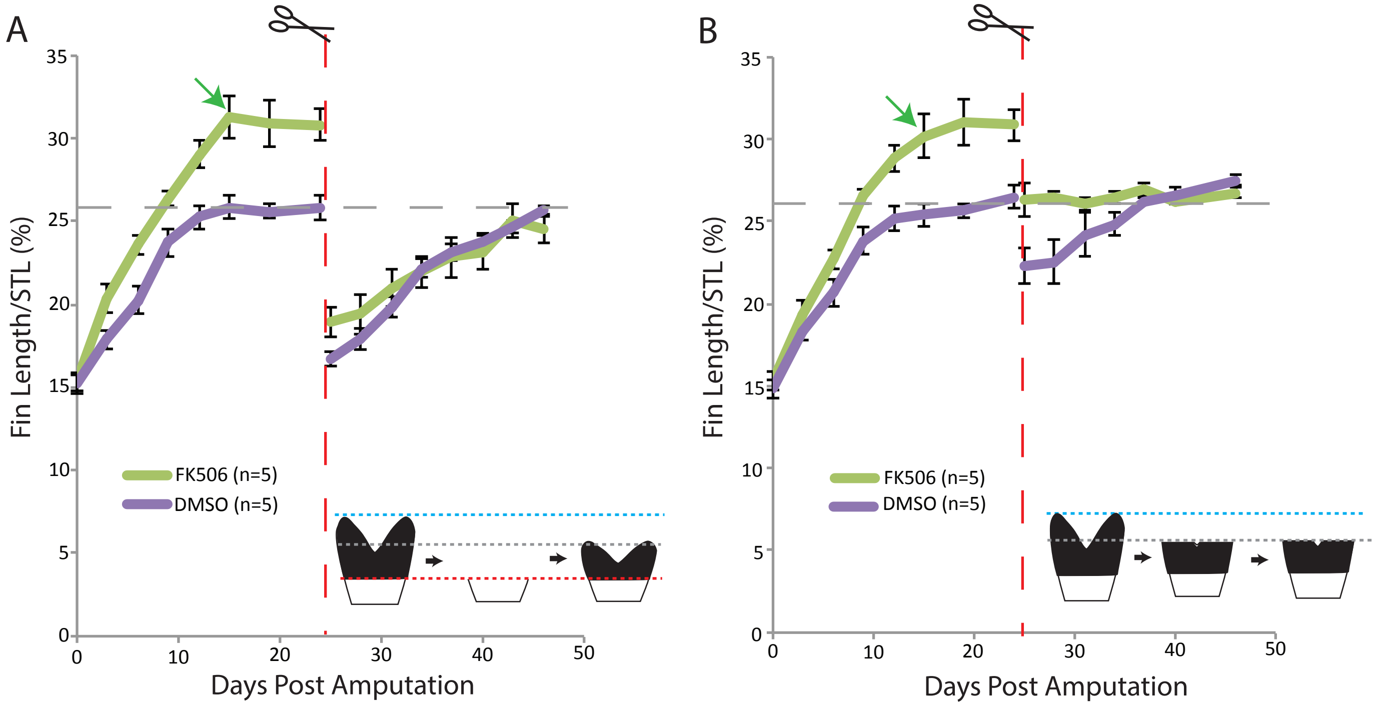
A
B
Fin Length/STL (%)
Days Post Amputation
FK506 (n=5)
DMSO (n=5)
0
5
10
15
20
25
30
35
0
10
20
30
40
50

# Figure S3

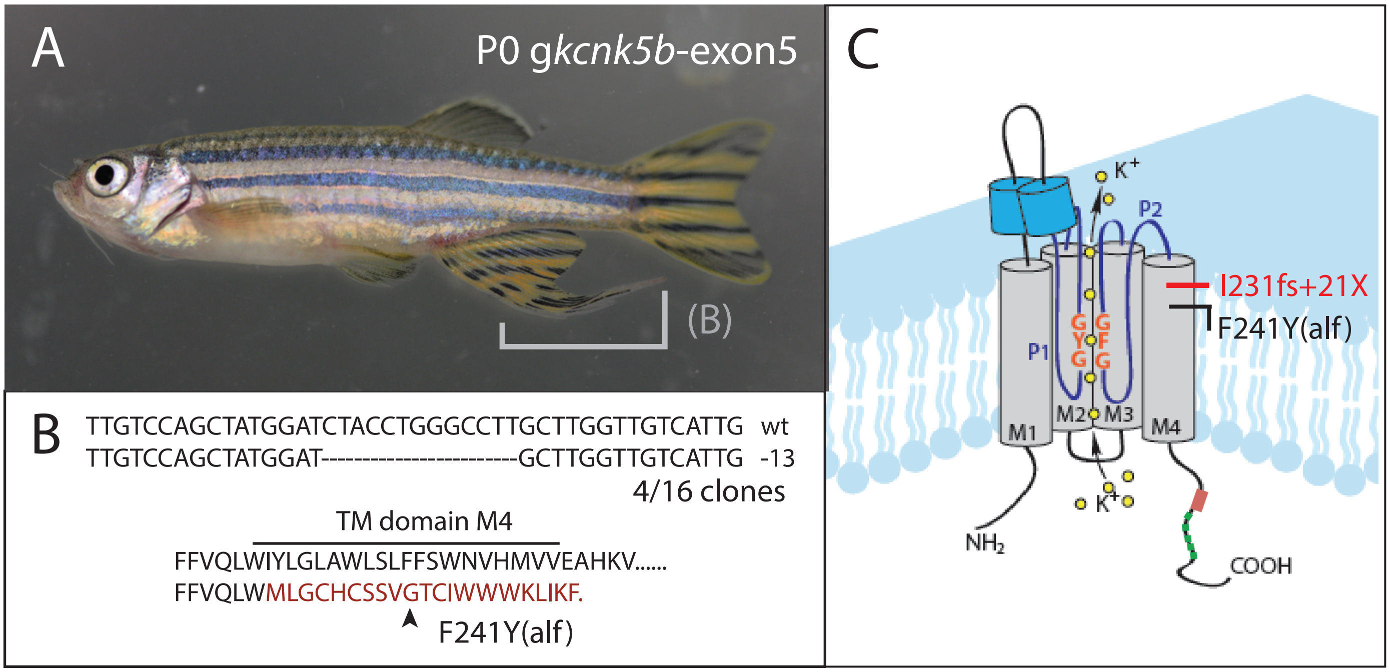
A
P0 gkcnk5b-exon5
(B)
B
TTGTCCAGCTATGGATCTACCTGGGCCTTGCTTGGTTGTCATTG wt
TTGTCCAGCTATGGAT-------------------------GCTTGGTTGTCATTG -13
4/16 clones
TM domain M4
FFVQLWIYLGLAWLSLFFSWNVHMVVEAHKV......
FFVQLWMLGCHCSSVGTCIWWWKLIKF.
F241Y(alf)
C
K+
P2
P1
G G
Y F
G G
M1
M2
M3
M4
I231fs+21X
F241Y(alf)
K+
NH2
COOH

# Figure S4

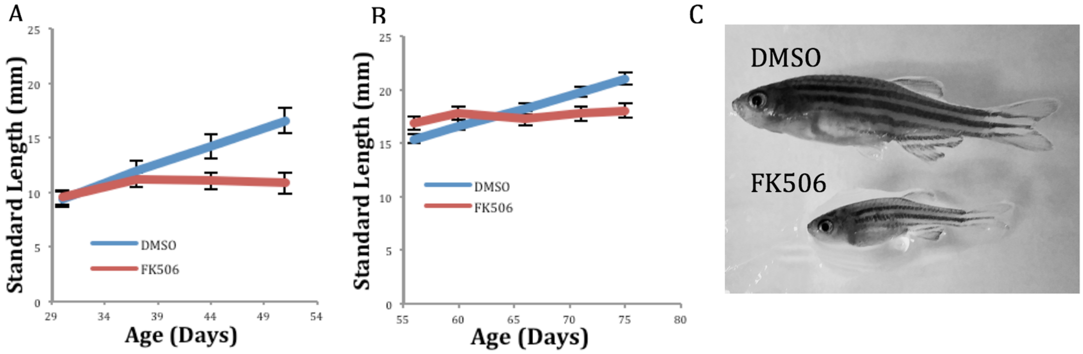
A
Standard Length (mm)
Age (Days)
0
5
10
15
20
25
29
34
39
44
49
54
DMSO
FK506
B
Standard Length (mm)
Age (Days)
0
5
10
15
20
25
55
60
65
70
75
80
DMSO
FK506
C
DMSO
FK506
